# Oral Administration of Nicotinamide Mononucleotide Is Safe and Efficiently Increases Blood Nicotinamide Adenine Dinucleotide Levels in Healthy Subjects

**DOI:** 10.3389/fnut.2022.868640

**Published:** 2022-04-11

**Authors:** Keisuke Okabe, Keisuke Yaku, Yoshiaki Uchida, Yuichiro Fukamizu, Toshiya Sato, Takanobu Sakurai, Kazuyuki Tobe, Takashi Nakagawa

**Affiliations:** ^1^Department of Molecular and Medical Pharmacology, Faculty of Medicine, University of Toyama, Toyama, Japan; ^2^First Department of Internal Medicine, Faculty of Medicine, University of Toyama, Toyama, Japan; ^3^Center for Clinical Research, Toyama University Hospital, University of Toyama, Toyama, Japan; ^4^Research and Development Division, Mitsubishi Corporation Life Sciences Limited, Chiyoda-ku, Japan; ^5^Research Center for Pre-disease Science, University of Toyama, Toyama, Japan

**Keywords:** NMN, NAD, aging, safety, clinical trial

## Abstract

Nicotinamide mononucleotide (NNM) is an orally bioavailable NAD^+^ precursor that has demonstrated beneficial effects against aging and aging-associated diseases in animal models. NMN is ultimately converted to NAD^+^, a redox cofactor that mediates many metabolic enzymes. NAD^+^ also serves as the substrate for poly(ADP-ribose) polymerase (PARP) and sirtuins, and regulates various biological processes, such as metabolism, DNA repair, gene expression, and stress responses. Previous mouse models showed that NMN administration can increase NAD^+^ in various organs and ameliorate aging-related diseases, such as obesity, diabetes, heart failure, stroke, kidney failure, and Alzheimer’s disease through NAD^+^-mediated pathways. However, evidence of its effect on humans is still scarce. In this study, we conducted a placebo-controlled, randomized, double blind, parallel-group trial to investigate the safety of orally administered NMN and its efficacy to increase NAD^+^ levels in thirty healthy subjects. Healthy volunteers received 250 mg/day of NMN (*n* = 15) or placebo (*n* = 15) for 12 weeks, and physiological and laboratory tests were performed during this period. In addition, NAD^+^ and its related metabolites in whole blood were examined. Oral supplementation of NMN for 12 weeks caused no abnormalities in physiological and laboratory tests, and no obvious adverse effects were observed. NAD^+^ levels in whole blood were significantly increased after NMN administration. We also observed the significant rise in nicotinic acid mononucleotide (NAMN) levels, but not in NMN. We also found that the increased amount of NAD^+^ was strongly correlated with pulse rate before the administration of NMN. These results suggest that oral administration of NMN is a safe and practical strategy to boost NAD^+^ levels in humans.

**Clinical Trial Registration:** JRCT [https://jrct.niph.go.jp/], identifier: [jRCTs041200034].

## Introduction

Population aging is one of the greatest global challenges as the proportion of the world’s population over 60 years will nearly double until 2050 (1). The inevitable process of aging leads to a decline in various physiological functions, including motor function, vision, hearing, immunity, and cognitive function. These declines further cause aging-related diseases, such as diabetes, cancer, and dementia. Therefore, delaying the process of aging is a strategy to combat aging-related diseases. However, aging is very complex phenomenon in which various genetic and environmental factors are involved. Among them, nutrition and metabolism are critical factors that are tightly related to the aging process (2). Excess or shortage of nutrients affects both aging and longevity. For instance, calorie restriction significantly suppresses the aging phenotypes and increases the lifespan in various organisms (3). Recently, nicotinamide adenine dinucleotide (NAD^+^) has attracted a lot of attention as an anti-aging molecule (4). NAD^+^ was discovered as a co-enzyme that mediates fermentation reactions over 100 years ago (5). It has been established that NAD^+^ mediates various redox reactions in energy metabolism pathways, such as glycolysis, tricarboxylic acid cycle, fatty acid oxidation, and mitochondrial oxidative phosphorylation (6). NAD^+^ also serves as a substrate for poly(ADP-ribose) polymerase (PARP), sirtuins, CD38, and SARM1 (4). PARP accumulates to a site of DNA single strand break (SSB) and induces auto-ADP-ribosylation that is in turn initiating the SSB repair process. Sirtuin is a NAD^+^-dependent deacetylase/deacylase, which removes the acetyl/acyl group from the lysine residues of the target protein, and regulates gene expression, stress response, and metabolism. Recently, NAD + has been shown to regulate mRNA stability through NAD^+^-capping in both bacterial and mammalian cells (7–9). Thus, NAD^+^ is now considered to mediate various aging processes through these functions. NAD^+^ levels in various tissues and cells decline with age and cause further aging phenotypes through the suppression of NAD^+^-mediated metabolism and the activities of PARP and sirtuin. Thus, this vicious cycle induces a further decline of NAD^+^ levels. NAD^+^ supplementation therapy is one of the interventions to break this cycle and delays the aging process by suppressing NAD^+^ decline with age.

NAD^+^ is synthesized from a variety of dietary precursors, such as L-tryptophan (Trp), nicotinic acid (NA), and nicotinamide (NAM), through *de novo*, Preiss-Handler, and salvage pathway, respectively (10–12). In particular, the salvage pathway is considered the most important pathway for the generation and maintenance of NAD^+^ levels in mammals. In the salvage pathway, nicotinamide phosphoribosyltransferase (Nampt) generates nicotinamide mononucleotide (NMN) from NAM followed by the conversion of NMN to NAD^+^ by nicotinamide mononucleotide adenylyltransferase (Nmnat). Although NAM is the authentic dietary source for NAD^+^ synthesis, Nampt is considered a rate-limiting enzyme. The generation of NAD^+^ from NAM seems inefficient. In addition, NAM is an inhibitor of sirtuins, and may cancel the sirtuin-mediated beneficial effects. Thus, nicotinamide riboside (NR) and NMN have been recognized as preferable NAD^+^ precursors for NAD^+^ supplementation therapy. The beneficial effects of NR and NMN have been reported in many animal models (13). NR is the most studied NAD^+^ precursor since its identification in 2004 (14). NR is a type of nucleoside that is found in cow’s but also in human’s milk. Oral administration of NR protects against diet-induced obesity and improves insulin sensitivity (15). NR supplementation has also been found to extend the lifespan of aged mice (16). The therapeutic effects of NR have also been shown in various mouse models, such as Alzheimer’s disease, non-alcoholic fatty liver disease, hearing loss, and mitochondrial myopathy (17–21). NMN is another orally available NAD^+^ precursor found in broccoli, edamame, cucumber, beef, and shrimp. Oral administration of NMN exhibits beneficial effects against diet- and aging-induced obesity and diabetes (22). Therefore, it has been suggested that long-term NMN supplementation ameliorates various age-related physiological declines in mice (23).

Recently, several clinical trials have investigated the effects of NR and NMN on humans (13, 24). Oral administration of NR has been demonstrated as safe and tolerable to human subjects. Apparent adverse effects, including flushing and liver damages that are frequently observed during niacin administration, were not observed. In addition, NAD^+^ levels were significantly elevated after NR administration (25–29). These results indicate that NR is a good NAD^+^ booster in healthy humans. However, the evidence to prove its therapeutic effects against aging and aging-related diseases is relatively limited. Several studies have reported that oral administration of NR could improve exercise performance, alter body composition, and enhance brown adipose tissue activity (30–32). In elderly people, 1 g of NR administration for 21 days was found to alter the mitochondrial-associated transcriptome in skeletal muscle and the circulating inflammatory cytokine profile; however, physiological parameters, such as body weight, blood pressure, lipid profile, and glucose metabolism, remained unaffected (33). Yet, other studies have demonstrated that oral administration of NR does not change mitochondrial functions in the skeletal muscle, and does not improve glucose metabolism in obese patients (34–36). The effects of NR have also been examined in patients with conditions such as heart failure, acute renal failure, and COVID-19 (37–39).

To date, three clinical trials have examined the safety and efficacy of NMN oral administration to humans (40–42). These studies demonstrated that oral administration of NMN is overall safe and tolerable. The first trial was a single arm and dose-escalation study that examined the clinical safety of NMN. The healthy volunteer received 100, 250, and 500 mg of NMN at one time. There were no apparent adverse effects, and no obvious change was observed during physiological examination and acquisition of laboratory data. Although NAD^+^ levels were not reported in this study, NAM-degraded metabolites, such as *N*-methyl-2-pyridone-5-carboxamide (2Py), *N*-methyl-4-pyridone-5-carboxamide (4Py), and *N*-methyl nicotinamide (MNAM), were significantly increased in urine. Another study demonstrated that oral administration of a 250 mg/day dose of NMN for 10 weeks could significantly increase NAD^+^ levels in PBMCs and improve insulin sensitivity in the skeletal muscle of obese pre-diabetic women. Finally, a more recent study showed that administration of NMN could enhance the aerobic capacity in amateur runners.

Although these studies have suggested that NMN supplementation is safe and can significantly increase NAD^+^ levels, more evidence is necessary to establish the safety and efficacy of NMN. In addition, data on NAD^+^ metabolism in humans during NMN supplementation are still missing. In the present study, we examined the safety of orally administrating NMN for 12 weeks in healthy volunteers. In addition, we investigated NAD^+^ metabolism during oral administration of NMN.

## Materials and Methods

### Ethical Approval and Informed Consent

This study was conducted according to the Declaration of Helsinki guidelines, and it was approved by the Clinical Research Review Board, University of Toyama (CRB4180013). The study was registered at jRCT (jRCTs041200034) before the participants were enrolled. Informed consent was obtained from each participant in person.

### Study Design, Randomization, and Intervention

The study was designed as a placebo-controlled, randomized, double blind, parallel-group trial. The sample size was estimated to be 26 with the following conditions; significance level: 0.10, the power of the test: 0.70, the expected proportions of the adverse events related to the intervention in both groups: 0.02, and the non-inferiority margin: 0.1. Assuming 10% of dropout, the actual sample size was determined to be thirty. Participants were examined at the screening center before starting administration (0-week), and at 4-, 8-, 12-week, and 4 weeks after finishing administration (16-week). Participants were randomly allocated to the NMN or the placebo group in a one to one ratio by C&C Qualitative Research Institute Inc. (Tokyo, Japan) based on the principal that the background and screening test results of participants were not biased between groups. The information of allocation was kept by C&C Qualitative Research Institute Inc., and the key open was done after all results were fixed. Participants in the NMN group were administered tablets containing 125 mg of NMN twice a day (total 250 mg of NMN per day) for 12 weeks, whereas patients in the placebo group were administered the tables without NMN in the same regimen. Both NMN and placebo were manufactured and supplied by Mitsubishi Corporation Life Sciences Limited (Tokyo, Japan).

### Study Participants

Forty-two healthy adult Japanese volunteers were recruited in this study, and were assessed for eligibility according to the inclusion and the exclusion criteria after their screening blood tests. The inclusion criteria were as follows: healthy Japanese volunteers between 20 and 65 years old, included by a medical doctor based on their clinical laboratory blood test results, which consisted of triglyceride (TG), LDL-cholesterol, fasting plasma glucose (FPG), HbA1c, AST, ALT, gamma-GTP, serum amylase, and serum creatinine. The exclusion criteria were as follows: (1) volunteers with past treatment history of malignant tumors, heart failure, myocardial infarction, or currently under treatment for atrial fibrillation, arrhythmia, hepatic dysfunction, renal dysfunction, cerebrovascular disorder, rheumatism, diabetes, dyslipidemia, hypertension, or other chronic diseases, (2) volunteers receiving medications (including Kampo medicine), (3) volunteers with known allergies to the medication or to the test food, (4) pregnant, unwilling to practice contraception during the study, or lactating females, (5) volunteers who did not qualify by the medical doctor. The enrolled participants were instructed not to change their lifestyle drastically and to avoid taking supplements or healthy food regularly.

### Primary and Secondary Outcomes

The primary outcome of this study was the safety assessment of NMN, which was evaluated by physical measurements, blood test, urine test, and subjective symptoms recorded in the diary of each participant. The secondary outcome involved an assessment of the NAD^+^-related metabolites and amino acids in whole blood.

### Evaluation of Safety, Tolerability, and Adherence

Subjective symptoms were recorded by each participant in a diary and were subsequently assessed at the visit every 4 weeks. The number of skipped tablets was also recorded in a diary. Participants were asked to report on any serious adverse events immediately. Laboratory data were evaluated every 4 weeks after starting the intervention so as to monitor potential adverse events.

### Clinical Laboratory Measurements

Laboratory measurements at screening included TG, LDL-cholesterol, FPG, HbA1c, AST, ALT, gamma-GTP, serum amylase, and serum creatinine. Laboratory measurements at 0-, 4-, 8-, 12-, and 16-week visit included white blood cell count, red blood cell count, hemoglobin, hematocrit, platelet count, MCV, MCH, MCHC, TG, total cholesterol, LDL-cholesterol, HDL-cholesterol, free fatty acid, acetoacetic acid, 3-hydroxyacetic acid, total ketone body, lipoprotein (a), FPG, HbA1c, immunoreactive insulin (IRI), AST, ALT, gamma-GTP, ALP, LDH, total protein, albumin, uric acid, urea nitrogen, creatinine, sodium, chloride, potassium, calcium, inorganic phosphorus, magnesium, and urine qualitative test (sugar, protein, urobilinogen, ketone body, pH, specific gravity, occult blood reaction, bilirubin). Blood was collected with fasting in the clinical laboratory department of the Toyama University Hospital between 8 and 9 a.m. HOMA-IR was calculated as follows: FPG (mg/dL) × IRI (μU/mL)/405. Furthermore, HOMA-β was calculated as follows: 360 × IRI (μU/mL)/(FPG [mg/dL] − 63). Blood samples for free fatty acid, acetoacetic acid, 3-hydroxyacetic acid, total ketone body, and lipoprotein (a) were sent to BML (BML, Inc. Tokyo, Japan) for measurements. Other blood tests were performed at the Toyama University Hospital.

### Measurement of Nicotinamide Adenine Dinucleotide Metabolome

Metabolite extraction and NAD metabolomics were performed according to a previously described method with slight modifications (43). Briefly, 10 μL of human whole blood was mixed with 490 μL of 50% MeOH. d4-NA and d4-NAM were added as internal standards at a concentration of 1 μM. The mixture was vortexed for 10 s to homogenize. For liquid-liquid extraction, 500 μL of chloroform was added and vortexed for 10 s. The mixture was centrifuged at 13,000 × *g* for 10 min at 4°C. The aqueous phase was transferred to a new tube, and liquid-liquid extraction was repeated. Following extraction, the aqueous phase was dried by using a SpeedVac SPD1010 (Thermo Fisher Scientific, Waltham, MA, United States). The dried sample was reconstituted in LC/MS grade water (FUJIFILM Wako Pure Chemical Corporation, Osaka, Japan) and filtered with 0.45 μm Millex filter unit (Merck Ltd. Tokyo, Japan). Metabolites were analyzed by the Agilent 6460 Triple Quad mass spectrometer coupled with Agilent 1290 HPLC system. The system was operated by MassHunter Workstation-Data Acquisition (Version B.05.00, Agilent Technologies, Santa Clara, CA, United States). Analytes were separated by Atlantis T3 Column (2.1 × 150 mm, 3-μm particle size, Waters) using mobile phase A (5 mM ammonium formate) and mobile phase B (methanol) with a flow rate of 150 μl/min and a column temperature of 40°C. The programmed mobile phase gradient was as follows: 0–10 min, 0%–70% B; 10–15 min, 70% B; 15–20 min, 0% B. Detection of labeled NAD^+^ metabolites was performed by using modulated transitions of m/z, equal fragmentor voltage, and equal collision energy with non-labeled NAD^+^ metabolites. Data were analyzed by MassHunter Workstation-Quantitative Analysis (Version B.05.00, Agilent technologies, Santa Clara, CA, United States) and quantifications were performed by using the standard curve obtained from the various concentrations of standard compounds.

### Measurements of Amino Acid Metabolome

Amino acid metabolomics was performed as described previously (44). After the extraction of metabolites, the samples were derivatized by incubating with *N*α-(5-Fluoro-2,4-dinitrophenyl)-L-leucinamide (FDLA). The derivatized samples were diluted with water and filtered before injection. Amino acid metabolites were also analyzed by the Agilent 6460 Triple Quad mass spectrometer coupled with Agilent 1290 HPLC system. HPLC separation of the amino acids was performed by using MG3 column (2.0 × 150 mm, 3-μm particle size, Osaka Soda, Japan) using mobile phase A (5 mM ammonium formate) and mobile phase B (methanol) with a flow rate of 150 μl/min and a column temperature of 40°C. The programmed mobile phase gradient was as follows: 0–10 min, 20–80% B; 10–15 min, 80% B; 15–15.01 min, 80–20% B. Data were analyzed by MassHunter Workstation-Quantitative Analysis (Version B.05.00, Agilent technologies, Santa Clara, CA, United States).

### Body Composition

Body composition, including soft lean mass, segmental lean mass (right arm, left arm, trunk, right leg, and right leg), skeletal muscle mass of whole body, %body fat, and bone mineral mass, was measured with InBody770 (InBody Japan Inc., Tokyo, Japan), which was a direct segmental multi-frequency bioelectrical impedance analyzer, at screening, 0-, and 12-week. The Skeletal Muscle Mass Index (SMI) (kg/m^2^) was calculated as follows: skeletal muscle mass (kg)/height^2^ (m^2^).

### Statistical Analysis

Statistical analyses were performed using JMP^®^ Pro 16.0.0. Data in the NMN group were compared with that of the placebo group at the corresponding time point using Mann–Whitney *U* test. Welch’s *t*-test was applied when normal distribution was not negated with Shapiro–Wilk test. Unpaired *t*-test was applied when normal distribution and homoscedasticity were not negated with Shapiro–Wilk test and Levene’s test, respectively. Correlation coefficients were calculated by using Pearson’s correlation coefficient formula. The definition of correlation was as follows: |R| < 0.2 very weak correlation, 0.2 ≦ |R| < 0.4 weak correlation, 0.4 ≦ |R| < 0.6 moderate correlation, 0.6 ≦ |R| < 0.8 strong correlation, 0.8 < |R| very strong correlation.

## Results

### Enrollment of Participants and Their Baseline Characteristics

**FIGURE 1:**
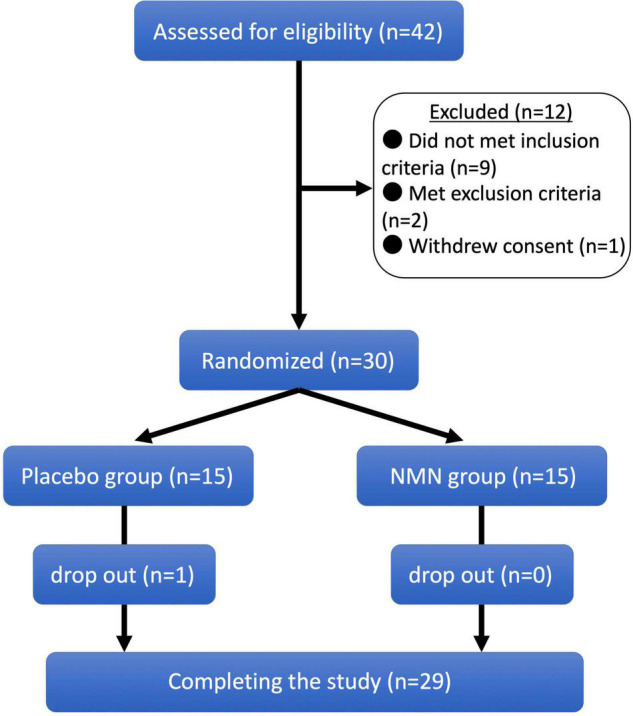
Clinical trial diagram. Clinical trial diagram based on the CONSORT. A total of 42 potential participants were screened, and 30 eligible participants were selected and randomized into nicotinamide mononucleotide (NMN) and placebo groups in a 1:1 ratio. One participant in placebo group was dropped out from the study at 8-week, and 29 participants completed the study.

Forty-two people aged between 22 and 64 years were assessed for eligibility in February 2021. Among them, 12 candidates were excluded because of the following reasons: nine candidates did not meet the inclusion criteria, two met the exclusion criteria, and one initially accepted and then withdrew the consent. Consequently, 15 participants were randomly allocated to the NMN group and 15 participants were allocated to the placebo group (Figure 1). The background characteristics of participants in the NMN and placebo groups at baseline are compared in Table 1. Age, sex, physical examinations, vital signs, and laboratory data (liver enzymes, amylase, lipid metabolism, and glucose metabolism) were comparable between the two groups.

**TABLE 1:** Background characteristics of the participants.

	Placebo (Mean ± SD)	NMN (Mean ± SD)	*p*-Value
Sex (female/male)	11/4	11/4	–
Age (years old)	43.9 ± 9.9	42.9 ± 12.0	0.92
Height (cm)	163.0 ± 8.5	162.3 ± 7.2	0.85
Body weight (kg)	56.2 ± 7.7	56.3 ± 8.6	0.97
BMI (kg/m^2^)	21.1 ± 2.1	21.3 ± 2.5	0.90
Body fat% (%)	25.6 ± 5.7	25.7 ± 5.0	0.92
Systolic BP (mmHg)	123.3 ± 10.7	121.1 ± 6.2	0.48
Diastolic BP (mmHg)	74.5 ± 7.5	74.3 ± 8.9	0.84
Pulse rate (bpm)	76.4 ± 8.9	76.2 ± 11.9	0.68
AST (IU/L)	19.7 ± 3.4	19.5 ± 5.2	0.66
ALT (IU/L)	17.3 ± 7.1	17.8 ± 10.8	0.56
γGT (IU/L)	17.7 ± 6.2	17.0 ± 9.4	0.31
Amylase (U/L)	92.1 ± 41.4	91.9 ± 29.0	0.82
Triglyceride (mg/dL)	66.5 ± 19.4	66.7 ± 19.2	0.95
LDL-cholesterol (mg/dL)	106.6 ± 17.3	105.2 ± 24.7	0.76
Glucose (mg/dL)	98.4 ± 6.5	97.5 ± 5.1	0.88
HbA1c (%)	5.38 ± 0.31	5.37 ± 0.23	0.98
Creatinine (mg/dL)	0.70 ± 0.14	0.73 ± 0.11	0.28

### Adverse Events Were Tolerable and Comparable Between the Nicotinamide Mononucleotide and the Placebo Groups

**FIGURE 2:**
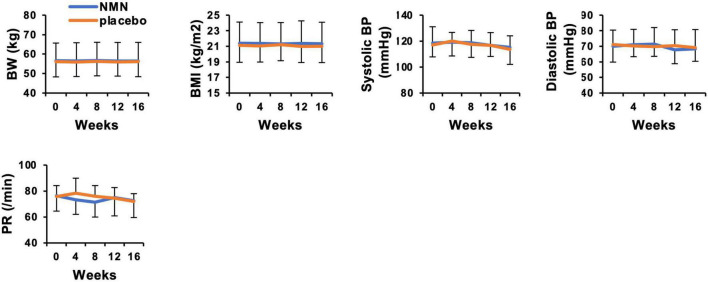
Nicotinamide mononucleotide have no effect on body weight, blood pressure, and pulse rate. Physical measurements were performed every 4 weeks. Orange represents placebo group (*n* = 15 at 0, 4, 8 weeks, *n* = 14 at 12, 16 weeks) and blue represents NMN group (*n* = 15). Data are represented as mean ± SD. BW, body weight; BMI, body mass index; BP, blood pressure; PR, pulse rate.

**FIGURE 3:**
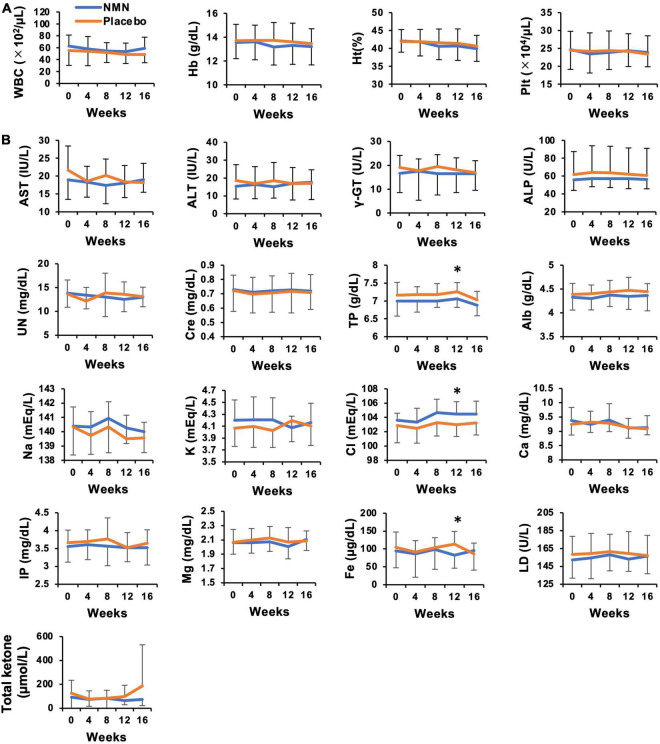
No adverse event was observed in laboratory data for safety evaluation of NMN oral administration. Hematological **(A)** and biochemical **(B)** blood tests were performed every 4 weeks. Orange represents placebo group (*n* = 15 at 0, 4, 8 weeks, *n* = 14 at 12, 16 weeks) and blue represents NMN group (*n* = 15). Data are represented as mean ± SD. WBC, white blood cells; Hb, hemoglobin; Ht, hematocrit; Plt, platelets; AST, aspartate aminotransferase; ALT, alanine aminotransferase; γ-GT, gamma-glutamyl transferase; ALP, alkaline phosphatase; UN, urine nitrogen; Cre, serum creatinine; TP, total protein; Alb, albumin; Na, serum sodium; K, serum potassium; Cl, serum chloride; Ca, serum calcium; Mg, serum magnesium; Fe, serum iron; IP, inorganic phosphorus; LD, lactate dehydrogenase. Asterisk means statistical significance: *p*-value < 0.05.

Participants took the NMN or placebo from March 2021 to May 2021 for 12 weeks. The consuming rate of NMN or placebo was 96.4% in the NMN group and 96.7% in the placebo group, respectively, during the trial period. All participants consumed more than 80% of NMN or placebo. There were seven (46.7%) and eight (53.3%) participants who complained about some symptoms in the placebo and the NMN group, respectively. There were no serious adverse events in either the placebo group or the NMN group (Table 2). However, there was one participant in the placebo group who discontinued due to gastrointestinal symptoms, i.e., a sense of hunger, abdominal distension, eructation, and flatus after started taking the placebo, which had continued for 56 days until quitting. In contrast, no participants discontinued in the NMN group. Adverse event due to the intervention occurred in one case in the placebo group, which was the same case described above, and in one case in the NMN group who complained of abdominal pain that started soon after taking NMN but spontaneously disappeared after 30 mins. There were two (13.3%) and six (40.0%) cases who complained of fever, joint pain, or fatigue starting within 1 day after the vaccination for COVID-19 in the placebo group and the NMN group, respectively (Table 2). Based on the diaries, there were six and seven cases who were vaccinated for COVID-19 in the placebo group and the NMN group, respectively, during the trial period. Other adverse events were considered to have no relevance to the placebo or the NMN. During the study period, body weight, BMI, systolic blood pressure, diastolic blood pressure, and pulse rate remained unchanged and presented no significant differences between the NMN and the placebo groups (Figure 2). Blood examinations were conducted at 0-week (before starting the administration of NMN or placebo), 4-, 8-, 12-week, and 16-week (4 weeks after finishing the administration of NMN or placebo). There was no significant difference between the NMN and placebo groups except for total protein, serum chloride, and serum iron at 12-weeks, which were within the normal range in both groups. Liver enzymes, renal function, other serum electrolytes, and complete blood count were within the normal range and presented no significant difference between the NMN and placebo groups (Figures 3A,B). There was one case with a considerably high level of ketone bodies without any symptoms; total ketone bodies 1,324 μmol/L, acetoacetic acid 310 μmol/L, and 3-hydroxybutyric acid 1,014 μmol/L because of scarce food intake on the day before the 16-week examination. This increase in ketone bodies was not regarded as an adverse event, but as a physiological reaction. Taken together, our data indicated that oral administration of 250 mg/day NMN for 12 weeks is both tolerable and safe in healthy people.

**TABLE 2:** Adverse events.

	Placebo	NMN
Any adverse event	7 (46.7%)	8 (53.3%)
Serious adverse event	0 (0.0%)	0 (0.0%)
Discontinuance of study	1 (6.7%)	0 (0.0%)
Adverse event due to intervention	1 (6.7%) *^1^	1 (6.7%) *^2^
Gastrointestinal symptoms*^3^	2 (13.3%)	3 (20.0%)
Fever, joint pain, or fatigue*^4^	2 (13.3%)	6 (40.0%)
Muscle pain*^4^	2 (13.3%)	2 (13.3%)
Upper respiratory tract symptoms*^5^	1 (6.7%)	1 (6.7%)
Hives	0 (0.0%)	1 (6.7%)
Headache	2 (13.3%)	0 (0.0%)
Keratitis, dry eye	1 (6.7%)	0 (0.0%)
Toothache	1 (6.7%)	0 (0.0%)

*^1^A sense of hunger, abdominal distension, eructation, and flatus.

*^2^Abdominal pain.

*^3^Abdominal pain, abdominal malaise, diarrhea, a sense of hunger, abdominal distension, eructation, or flatus.

*^4^Side effect of vaccination for COVID-19 is suspected.

*^5^Cough, phlegm, sore throat, nasal discharge.

### Clinical Metabolic Effects of Nicotinamide Mononucleotide Administration Were Not Apparent for Healthy Volunteers

**FIGURE 4:**
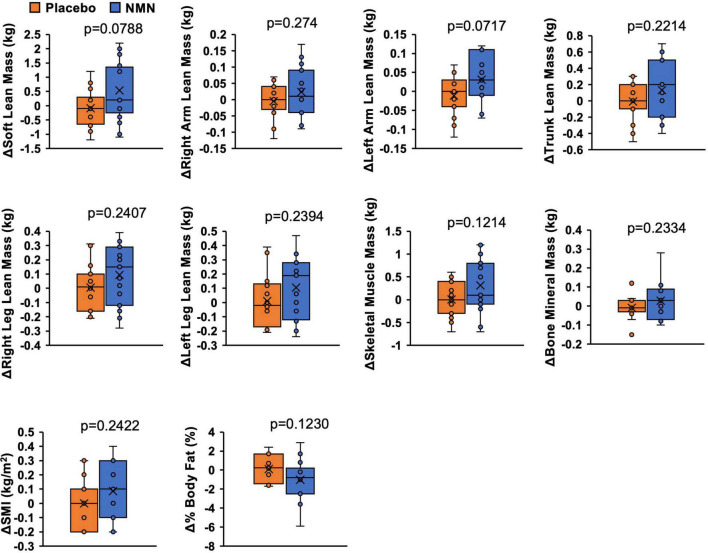
Amount of change of body composition implied increased muscle mass and reduced body fat. Body composition was measured with InBody770 at 0- and 12-week. Value at 0-week was subtracted from value at 12-week to calculate amount of change. Orange represents placebo group (*n* = 14) and blue represents NMN group (*n* = 15). Data are represented as mean ± SD. SMI, skeletal muscle mass index.

**FIGURE 5:**
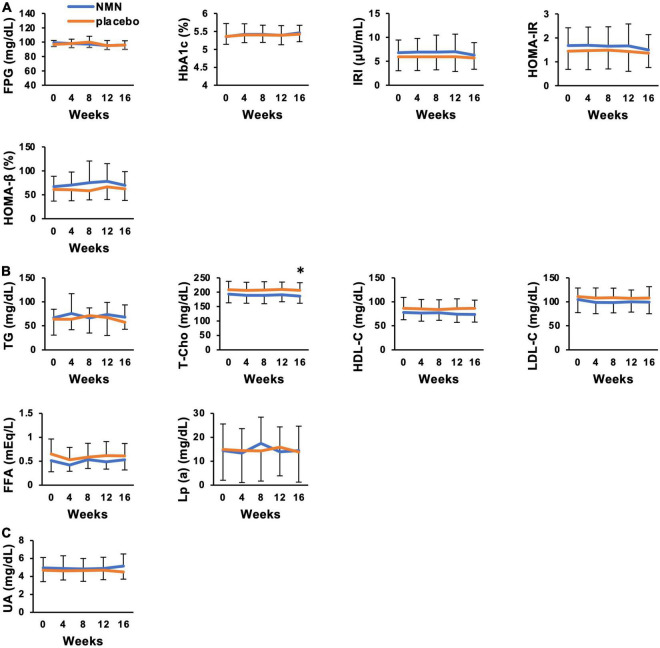
Clinical metabolic effects of NMN oral administration were not apparent. Blood tests were performed every 4 weeks to evaluate glucose metabolism **(A)**, lipid metabolism **(B)**, and uric acid level **(C)**. Orange represents placebo group (*n* = 15 at 0, 4, 8 weeks, *n* = 14 at 12, 16 weeks) and blue represents NMN group (*n* = 15). Data are represented as mean ± SD. FPG, fasting plasma glucose; HbA1c, hemoglobin A1c; IRI, immunoreactive insulin; HOMA-IR, homeostasis model assessment-insulin resistance; HOMA-β, homeostasis model assessment-beta cell function; TG, triglyceride; T-cho, total cholesterol; HDL-C, high density lipoprotein-cholesterol; LDL-C, low density lipoprotein-cholesterol; FFA, free fatty acid; UA, uric acid; Lp (a), lipoprotein (a). Asterisk means statistical significance: *p*-value < 0.05.

Lean mass, skeletal muscle mass, SMI, bone mineral mass, and %body fat were assessed by a direct segmental multi-frequency bioelectrical impedance analyzer, InBody770. Although there was no statistically significant difference between the two groups, the amount of change in soft lean mass (*p* = 0.0788), left arm lean mass (*p* = 0.0717), skeletal muscle mass (*p* = 0.1214), and %body fat (*p* = 0.1230) suggested increased skeletal muscle mass and reduced body fat in the NMN group (Figure 4). Clinical effects of NMN administration on glucose metabolism, lipid metabolism, and uric acid were examined by blood examinations performed every 4 weeks (Figures 5A–C). All these markers were within the normal range and did not exhibit significant differences between the NMN group and the placebo group during the study period of 16 weeks.

### Levels of Nicotinamide Adenine Dinucleotide and Nicotinic Acid Mononucleotide in Whole Blood Were Significantly Increased After Nicotinamide Mononucleotide Administration

**FIGURE 6:**
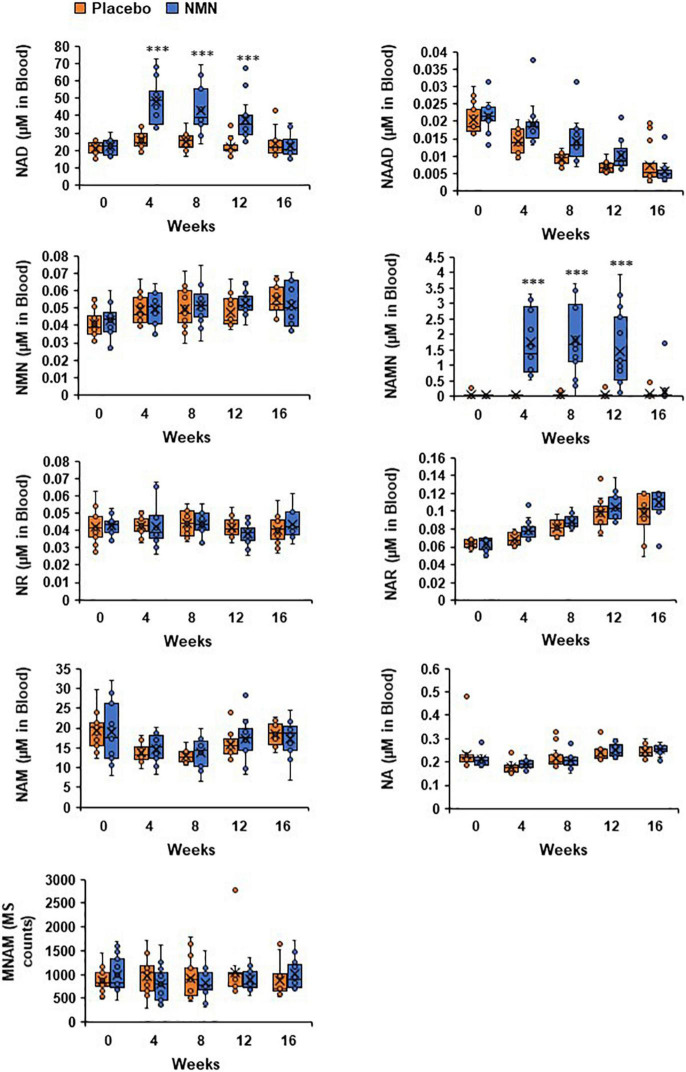
Levels of NAD^+^ and NAMN in blood were increased by oral administration of NMN. NAD metabolome in blood was measured every 4 weeks. NAD metabolome in each time point was showed in box plots. In the box plots, the median is indicated by the line within the boxes. The lower and upper boundaries of the boxes indicated 25th and 75th percentiles. The upper and lower lines above and below the boxes represent the whiskers. Orange boxes represent placebo group (*n* = 15 at 0, 4, 8 weeks, *n* = 14 at 12, 16 weeks) and blue boxes represents NMN group (*n* = 15). Levels of MNAM were calculated by integrating peak area of each chromatogram (MS counts). Three asterisks mean statistical significance: *p*-value < 0.001.

Measurements of NAD metabolome in whole blood were conducted at 0-week (before starting the administration of NMN or placebo), 4-, 8-, 12-, and 16-week (4 weeks after finishing the administration of NMN or placebo). In this study, the amounts of NAD^+^, NMN, NAM, NA, NAMN, NAR, NAAD, and MNAM were measured by LC/MS at the University of Toyama. The whole blood NAD metabolome levels at the baseline (0-week) were comparable between the NMN and placebo groups (Figure 6). Following oral administration of NMN or placebo, NAD^+^ levels were significantly increased in the NMN-treated group at 4-, 8-, 12 weeks, and then returned to basal levels after 16 weeks (Figure 6). This result was also confirmed by individual data that showed a bridge-shaped transition of NAD^+^ levels in the NMN-treated group (Figure 6). In contrast, NMN levels were not increased after NMN or placebo administration, and were comparable between the two groups (Figure 6). Additionally, NR and NAM levels were not significantly changed between the two groups (Figure 6). Remarkably, NAMN levels were significantly increased in the NMN group at 4-, 8-, and 12-weeks, and then returned to basal levels at 16 weeks (Figure 6). Individual data of NAMN levels in the NMN group also showed a bridge-shaped transition similar to that of the NAD^+^ levels. In this study, NA, NAR, NAAD, and MNAM levels remained unchanged between the two groups at any time points (Figure 6).

### Levels of Amino Acids in Whole Blood Were Not Changed After Nicotinamide Mononucleotide Administration

**FIGURE 7:**
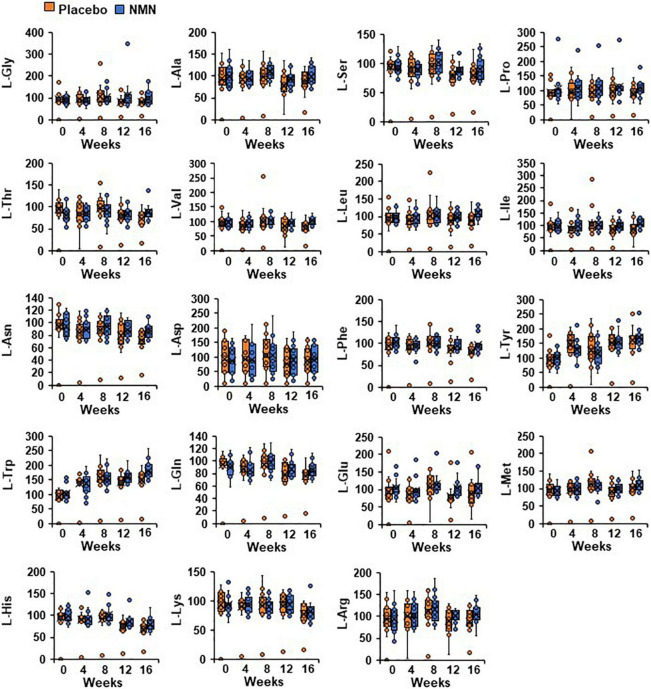
Oral NMN treatment had little effect on blood amino acid levels. Amino acid metabolome in blood was measured every 4 weeks. Amino acid metabolome in each time point was showed in box plots. In the box plots, the median is indicated by the line within the boxes. The lower and upper boundaries of the boxes indicated 25th and 75th percentiles. The upper and lower lines above and below the boxes represent the whiskers. Orange boxes represent placebo group (*n* = 15 at 0, 4, 8 weeks, *n* = 14 at 12, 16 weeks) and blue boxes represents NMN group (*n* = 15).

Nicotinamide adenine dinucleotide is a key molecule associated with various metabolic pathways, including amino acid metabolism. Additionally, serum amino acids levels, especially branched-chain amino acids (BCAA), have been reported to be related with the development of insulin resistance and diabetes (45–48). Therefore, we measured amino acid metabolome in whole blood to investigate the effect of orally administrated NMN on the metabolic pathways. The basal amino acid levels were comparable between the two groups (Figure 7). Furthermore, there were no changes in the amino acid levels, including BCAA between and within the groups at any time points (Figure 7).

### Correlations Between Individual Parameters and the Increase of Nicotinamide Adenine Dinucleotide After Administration of Nicotinamide Mononucleotide

**FIGURE 8:**
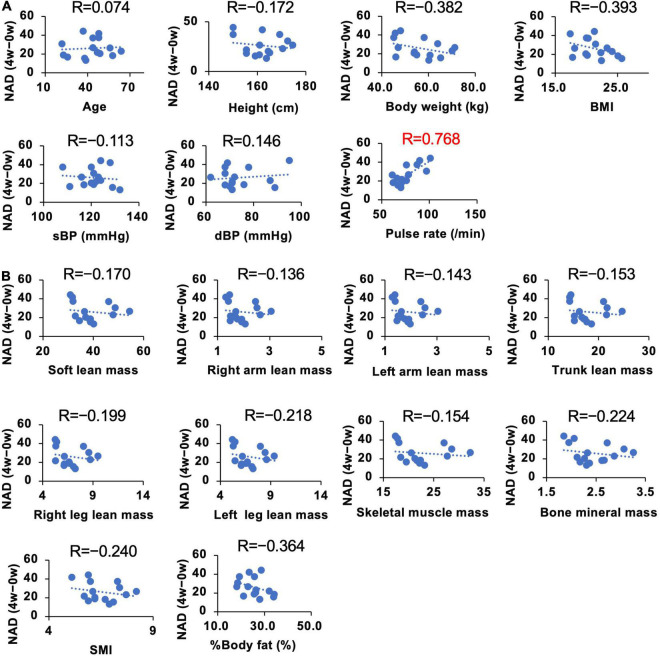
Correlation between individual basal physical parameters and amounts of increased NAD^+^ levels. The Y axis of each panel was fixed as the difference of blood NAD levels in 4 weeks. NAD levels at 0 weeks were subtracted from NAD levels at 4 weeks to calculate the change. Basal physical parameters **(A)** and body composition **(B)** at 0 weeks were compared with the difference of NAD at 4 weeks. Correlation coefficients were calculated by using Pearson’s correlation coefficient formula and indicated as R on the top of each panel.

**FIGURE 9:**
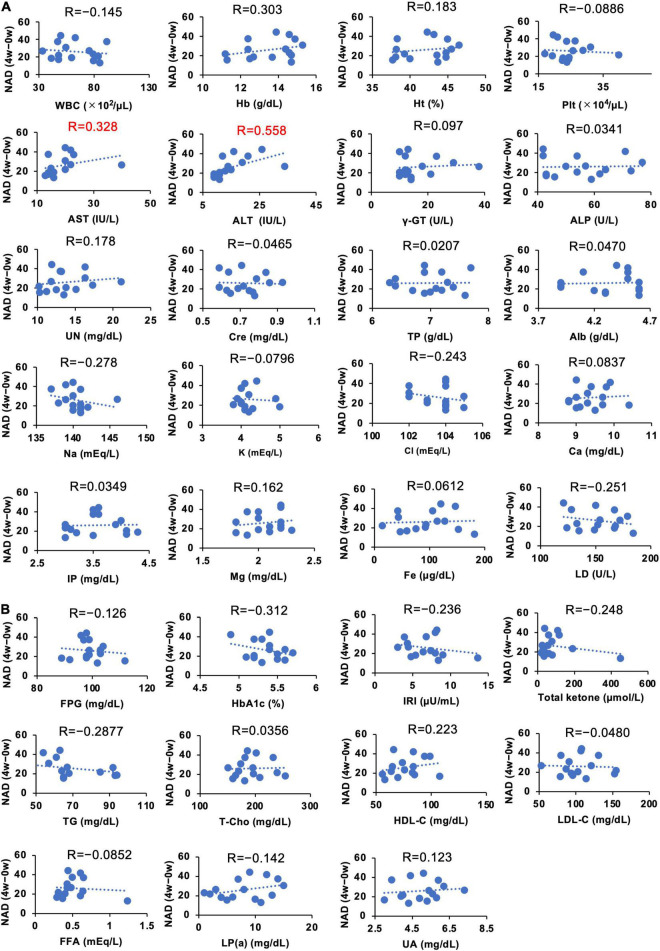
Correlation between individual basal blood parameters and amounts of increased NAD^+^ levels. The Y axis of each panel was fixed as the difference of blood NAD levels in 4 weeks. NAD levels at 0 weeks were subtracted from NAD levels at 4 weeks to calculate the change. Hematological and biochemical blood parameters **(A)** and blood parameters related to energy metabolism **(B)** at 0 weeks were compared with the difference of NAD at 4 weeks. Correlation coefficients were calculated by using Pearson’s correlation coefficient formula and indicated as R on the top of each panel.

We demonstrated that the administration of NMN significantly increased whole blood NAD^+^ levels. However, the individual susceptibility to NAD^+^ increase upon oral NMN administration may vary (Figure 6). To investigate individual parameters that may predict a higher sensitivity to the NMN treatment, we assessed the correlations between individual basal parameters and the increase of NAD^+^ levels in the NMN-treated group (Figures 8A,B, 9A,B). Although most of physiological parameters, including age, body weight, BMI, and body compositions at 0-week, showed no or weak correlations (|R| < 0.4), the pulse rate showed a strong positive correlation with an increase in NAD^+^ (R = 0.768; Figure 8A). Among the laboratory tests, ALT was the only parameter that showed a moderate positive correlation with an increase in NAD^+^ levels (*R* = 0.558; Figure 9A). In addition, AST revealed a weak positive correlation coefficient (*R* = 0.328; Figure 9A).

## Discussion

The present study investigated the safety of orally administered NMN in healthy participants. We also examined the NAD^+^ metabolism and amino acid metabolism during NMN administration. We demonstrated that taking 250 mg of NMN every day for 12 weeks is a safe and well-tolerated practice in healthy individuals. In addition, the NAD^+^ levels in whole blood were significantly increased in the NMN-treated group. These results suggest that the oral administration of NMN is safe and can be a practical strategy to boost NAD^+^ levels in humans.

The primary outcome of this study was to assess the safety of NMN oral administration. No abnormalities were observed in the laboratory data, including liver function markers, AST, ALT, and γ-GTP. Although high dosage of NA, around 2 g per day, are used for the treatment of dyslipidemia in clinical practice, the amount of NMN in this study was significantly smaller and not harmful to liver. Another study examined 500 mg of NMN administration at a single dosage, but no significant increase was observed in the levels of AST and ALT (40). As NMN is a nucleotide, we also examined the levels of uric acid (UA), and we found that they were stable during administration of NMN. Thus, NMN administration may not disturb UA metabolism at least in healthy people. NA is a drug for dyslipidemia that decreases LDL-cholesterol and triglyceride (TG) serum levels (49, 50). However, the TG, LDL-cholesterol, HDL-cholesterol, and Total-cholesterol levels remained unchanged both in the placebo and the NMN groups. The subjects in this study were healthy volunteers, and the levels of these lipids were almost within normal range, and no significant changes were observed during NMN administration. Flushing is a commonly observed side-effect in NA-treated patients (49). In this study, no such complaint was observed in both the placebo and the NMN-treated groups. Only gastrointestinal symptoms were the observed adverse effects associated with this intervention in both placebo and NMN- treated groups (Table 2). One person treated with placebo was dropped off from the study at 8 weeks due to the continuous abdominal bloating. Although one participant treated with NMN also had a mild stomach discomfort right after taking the NMN, this symptom disappeared without any medications. In this study, the vaccination for COVID-19 was allowed. Two of six in the placebo group and six of seven in the NMN group subjects complained the adverse reactions, such as fever, joint pain, or fatigue, after the vaccination (Table 2). Although there might be the trend of higher incidence in the NMN group, the difference between the groups was not statistically significant (*p* = 0.10 in Fisher’s exact test). It is suggested that viral infection depleted the NAD^+^ levels and blunted the anti-viral effects (51–53). Thus, NMN administration increases NAD^+^ levels and may boost innate immunity during COVID-19 infection or vaccination. Taken together, we concluded that oral administration of 250 mg NMN for 12 weeks is safe and well-tolerated.

In this study, we administrated 250 mg of NMM per day by dividing to 125 mg twice in the morning and evening. In a previous animal study, NMN was administered at 300 mg/kg per day for 12 months (23). However, the increase in NAD^+^ levels in tissues was marginal. Although 250 mg/day is significantly less compared to the dosage administered in mice, considering dose conversion from animal to human studies (approximately 1.2 g/day), 250 mg of NMM per day was an adequate dosage to increase and sustain the NAD^+^ levels in blood. Furthermore, NMN was fed with drinking water or food in the animal studies, whereas human took NMN as tablets twice per day. Perhaps the difference in the ways of taking NMN may attribute to the elevation of NAD^+^ levels in blood.

Human clinical trials have shown that NR can boost NAD^+^ levels in peripheral blood mononuclear cells (PBMC) (25, 28) and whole blood (29, 33, 37). In addition, NAD^+^ levels in urine were also increased by NR administration (35). Thus, it has been widely shown that NR can increase NAD^+^ levels in human blood. In contrast, only one study has reported the effect of NMN on NAD^+^ levels in human subjects. This study reported the increase of NAD^+^ levels in PBMC after administration of NMN at 250 mg/day for 10 weeks (41). In line with the findings of that study, our study also demonstrated that administration of NMN at 250 mg/day could significantly increase NAD^+^ levels in the whole blood. In addition, our study revealed the time course change of NAD^+^ levels. Although NAD^+^ levels were significantly increased at 12 weeks, the highest level was observed at 4 weeks. It is possible that an adaptation to higher NAD^+^ levels had occurred, and certain NAD synthesis enzymes, such as Nmnat, were downregulated. It is important to investigate whether longer NMN administrations periods (greater than 1 year) can still sustain increased NAD^+^ levels.

We also observed the drastic increase in NAMN levels after administration of NMN. The significant increase in deamidated NAD^+^-related metabolites, such as NAAD and NAR, has also been observed in other studies that administered NR to human subjects (25, 28, 35). It has been reported that orally administered NAM was converted to NA through deamidation by intestinal microbiota, and was absorbed from large intestine as NA (54). Recently, we demonstrated that orally administered NR was cleaved to NAM by BST1 followed by conversion to NA by microbiota (55). The absorbed NA contributed to NAD^+^ synthesis through the Preiss-Handler pathway generating NAMN as an intermediate. Therefore, it is possible that orally administered NMN is also converted to NA by microbiota in human subjects. Indeed, both NR and NMN were reported to fail to increase NAD^+^ levels in skeletal muscles, which lack the Preiss-Handler pathway (30, 33, 36, 41). It is very important to clarify the metabolic pathways of orally administered NMN in humans in order to maximize the efficiency of NMN supplementations.

It is clearly demonstrated that NMN can significantly increase NAD^+^ levels in whole blood, but the extent of this increase may vary among individuals. Thus, we analyzed the correlations between individual parameters and the increased amount of NAD^+^ levels after NMN administration. Because we used a fixed dosage of NMN for all participants in this study, the body weight and age were assumed as correlated factors. However, physical parameters, such as body weight and body composition, revealed a weak correlation. In contrast, pulse rate exhibited a strong positive correlation with the increase in NAD^+^ levels. The exact reason is unclear, but pulse rate may be related to energy expenditure (56), and it is possible that pulse rate is a confounding factor of energy expenditure. However, further studies are necessary for the precise interpretation of this result. Several blood parameters, such as Hb, Na, LD, Total ketone, HbA1c, TG, and HDL, also showed a weak correlation with the increase in NAD^+^ levels. Among these parameters, ALT showed the highest correlation. Liver is the most important organ to generate NAD^+^ in humans. In particular, deamidated NAD synthesis pathways, such as *de novo* and Preiss-Handler pathway (57), were most active in the liver. In our study, we observed the significant rise in NAMN levels, an intermediate of deamidated NAD synthesis pathway. It is possible that certain liver functions may correlate with NAD synthesis after NMN oral administration.

## Study Limitations

In this study, 30 healthy volunteers were enrolled. Although this study verified the significant and sufficient increase in NAD^+^ levels in whole blood, further evaluations are necessary in a larger sample size to confirm the correlations among the individual parameters and the increased amount of NAD^+^ after NMN administration. NAD^+^ levels in other tissues, such as skeletal muscle, were not examined. Many studies have failed to increase NAD^+^ levels in the skeletal muscle, and thus it is important to investigate the effect of NMN administration on other tissues and organs. In addition, this study did not demonstrate the consequences of increased NAD^+^. Alternation of NAD^+^-mediated metabolism and physiological activities should be examined in healthy and non-healthy subjects.

## Conclusion

The present study demonstrated that oral NMN administration of 250 mg/day can significantly increase and sustain the levels of NAD^+^ in whole blood at 4-weeks until the end of administration without any apparent adverse effects. In addition, we found that the HR is strongly correlated with the increase in NAD^+^. Thus, oral administration of NMN can be a practical strategy to boost NAD^+^ levels in humans.

## Data Availability Statement

The raw data supporting the conclusions of this article will be made available by the authors, without undue reservation.

## Ethics Statement

The studies involving human participants were reviewed and approved by the Clinical Research Review Board, University of Toyama (CRB4180013). The patients/participants provided their written informed consent to participate in this study.

## Author Contributions

KO, KY, and TN conceived the study, interpreted the data, and wrote the manuscript. KO and TN designed and conducted the clinical trials. YU, YF, TaS, and ToS manufactured NMN and the placebo. KT set up the analysis tools for body compositions. KY performed metabolome analysis. KO and KY conducted statistical analysis. All authors read and approved the final manuscript.

## Conflict of Interest

YU, YF, TaS, and ToS were employees of Mitsubishi Corporation Life Sciences Limited and prepared NMN and the placebo. The remaining authors declare that the research was conducted in the absence of any commercial or financial relationships that could be construed as a potential conflict of interest.

## Publisher’s Note

All claims expressed in this article are solely those of the authors and do not necessarily represent those of their affiliated organizations, or those of the publisher, the editors and the reviewers. Any product that may be evaluated in this article, or claim that may be made by its manufacturer, is not guaranteed or endorsed by the publisher.
